# Improving Survival in Cardiogenic Shock—A Propensity Score-Matched Analysis of the Impact of an Institutional Allocation Protocol to Short-Term Mechanical Circulatory Support

**DOI:** 10.3390/life12111931

**Published:** 2022-11-19

**Authors:** Sascha Ott, Daniel Lewin, Gaik Nersesian, Julia Stein, Isabell A. Just, Matthias Hommel, Felix Schoenrath, Christoph T. Starck, Benjamin O’Brien, Volkmar Falk, Evgenij Potapov, Pia Lanmueller

**Affiliations:** 1 Department of Cardiac Anesthesiology and Intensive Care Medicine, German Heart Center Berlin, 13353 Berlin, Germany; 2 Department of Cardiac Anesthesiology and Intensive Care Medicine, Charité—Universitätsmedizin Berlin, 13353 Berlin, Germany; 3 DZHK (German Centre for Cardiovascular Research), Partner Site Berlin, 13353 Berlin, Germany; 4 Department of Cardiothoracic and Vascular Surgery, German Heart Center Berlin, 13353 Berlin, Germany; 5 Department of Cardiothoracic Surgery, Charité—Universitätsmedizin Berlin, 13353 Berlin, Germany; 6 Institute (STI) of Cardiovascular Perfusion, Steinbeis University Berlin, 13353 Berlin, Germany; 7 St Bartholomew’s Hospital and Barts Heart Centre, Department of Perioperative Medicine, London EC1A 7BE, UK; 8 Berlin Institute of Health, Charité—Universitätsmedizin Berlin, 10178 Berlin, Germany; 9 Translational Cardiovascular Technologies, Institute of Translational Medicine, Department of Health Sciences and Technology, Swiss Federal Institute of Technology, 8092 Zürich, Switzerland

**Keywords:** cardiogenic shock, mechanical circulatory support, standard operating procedure, extracorporeal life support, percutaneous microaxial flow pump technology, LV unloading

## Abstract

Temporary mechanical circulatory support (tMCS) is a life-saving treatment option for patients in cardiogenic shock (CS), but many aspects such as patient selection, initiation threshold and optimal modality selection remain unclear. This study describes a standard operating procedure (SOP) for tMCS allocation for CS patients and presents outcome data before and after implementation. Data from 421 patients treated for CS with tMCS between 2018 and 2021 were analyzed. In 2019, we implemented a new SOP for allocating CS patients to tMCS modalities. The association between the time of SOP implementation and the 30-day and 1-year survival as well as hospital discharge was evaluated. Of the 421 patients included, 189 were treated before (pre-SOP group) and 232 after implementation of the new SOP (SOP group). Causes of CS included acute myocardial infarction (n = 80, 19.0%), acute-on-chronic heart failure in patients with dilated or chronic ischemic heart failure (n = 139, 33.0%), valvular cardiomyopathy (n = 14, 3.3%) and myocarditis (n = 5, 1.2%); 102 patients suffered from postcardiotomy CS (24.2%). The SOP group was further divided into an SOP-adherent (SOP-A) and a non-SOP-adherent group (SOP-NA). The hospital discharge rate was higher in the SOP group (41.7% vs. 29.7%), and treating patients according to the SOP was associated with an improved 30-day survival (56.9% vs. 38.9%, OR 2.21, 95% CI 1.01–4.80, *p* = 0.044). Patient allocation according to the presented SOP significantly improved 30-day survival.

## 1. Introduction

Despite significant progress in the diagnosis of cardiogenic shock (CS) and our understanding of the pathophysiology and management strategies of CS, morbidity and mortality remain high [1,2]. CS forms the common final path of different etiologies with acute myocardial infarction-related cardiogenic shock (AMICS) as one of the most frequent causal factors [3]. Acute-on-chronic heart failure in the presence of dilated or ischemic heart failure, valvular cardiomyopathy, myocarditis and other underlying cardiac pathologies may lead to CS [4]. Postcardiotomy cardiogenic shock (PCCS) following cardiac surgery has a special significance due to the very heterogeneous cohort, underlying causes and the particularly high mortality [5].

CS commonly presents as a progressing syndrome with rapid deterioration of the patient’s condition, associated with a significant increase in morbidity and mortality as shock status escalates [6]. In this context, 30-day mortality rates above 50% in AMICS patients and up to 75% in PCCS patients are reported [5,7]. For patients classified as stage E according to the Society for Cardiovascular Angiography and Interventions (SCAI), Schrage et al. recently showed a 30-day mortality rate of nearly 80% [6]. Until recently, temporary mechanical circulatory support (tMCS) devices were seen as a strategy of last resort. In the past decade, tMCS has shifted more into focus, and its use in CS was upgraded in the latest ESC Guidelines for the diagnosis and treatment of acute and chronic heart failure [8]. Due to the technological and procedural advances over the last few years, it is possible to bridge an increasing number of patients to recovery, transplant or durable MCS [9,10,11]. Nevertheless, evidence remains sparse and there is still some uncertainty about numerous aspects, such as patient and device selection and the timing and duration of tMCS. Aside from the necessity for randomized controlled trials to answer these questions, specific protocols updated with the latest developments in tMCS, stipulating defined patient and device selection criteria, are reasonable strategies to improve patient outcomes [12,13]. In particular, the indications for ECLS, percutaneous microaxial flow pump technology and the combination of both support systems have evolved over the past years.

In this study, we describe the implementation of a new standard operating procedure (SOP) for the application of tMCS in patients suffering from CS and the impact of this standardized approach on outcomes.

## 2. Materials and Methods

This study is a retrospective analysis of patients suffering from CS of all etiologies that were treated with tMCS at our institution between 2018 and 2021. The tMCS SOP was introduced in October 2019. The study was conducted in accordance with the Declaration of Helsinki and approved by the Institutional Ethics Committee (application number: EA2/196/21). The Institutional Ethics Committee waived the requirement for informed consent, due to the retrospective nature of the analysis and since only anonymized data were collected and analyzed. 

### 2.1. Patient Selection

Data from 451 consecutive patients suffering from CS who were treated with tMCS at our institution between January 2018 and May 2021 were extracted from the electronic health record. CS was defined according to ESC guidelines for the diagnosis and treatment of acute and chronic heart failure [14]. Exclusion criteria for this study were patients aged <18 years, patients with congenital heart disease and patients with multiple MCS runs. PCCS patients were not excluded.

### 2.2. Observed Outcomes

The observed outcomes of the study were 30-day survival, hospital discharge and 1-year survival. 

### 2.3. Data Collection

Demographic, clinical and hemodynamic data as well as laboratory values before tMCS implantation were collected from the electronic patient records. Data for 1-year survival were requested from a national registry database (Berlin civil registry office). The vasoactive–inotropic score (VIS) was calculated before MCS implantation using the following formula: dopamine + dobutamine + milrinone (×10) + epinephrine (×100) + norepinephrine (×100) (all µg/kg/min) + vasopressin (×10,000) (IU/kg/min) [15].

### 2.4. Statistical Analysis

Continuous variables are summarized as mean and standard deviation (SD), or as median and interquartile range [Q1, Q3] in the case of non-normal data. For categorical variables, numbers and percentages are presented. Patient groups were compared using Student’s *t*-test for normally distributed continuous data and the Mann–Whitney-U test for non-normally distributed continuous data. For categorical data, Chi² tests with Yates’ continuity correction were used. We used standardized mean difference (SMD) as a measure of imbalances between the two patient groups. 

Due to imbalances in confounding variables between the pre- and post-SOP patient groups, we used propensity score matching. We calculated the propensity score by logistic regression with predefined variables (age, gender, BMI, diabetes, chronic kidney disease, AMI, previous heart surgery, acute-on-chronic cardiomyopathy, previous episode of cardiac arrest/resuscitation, and MAP before MCS). We performed 1:1 propensity score matching using the nearest-neighbor algorithm without replacement and with a caliper width of 0.4. The balance of covariates was considered satisfactory for a standardized mean difference (SMD) of 0.2.

Survival was evaluated by Kaplan–Meier estimates with 95% confidence intervals (CIs). The risk of all-cause mortality in the pre-SOP group compared to the post-SOP group was estimated using a stratified Cox regression on the matched data, and hazard ratios (HRs) with 95% CIs are given.

To estimate the effect of the implementation of the SOP or adherence on binary outcomes, logistic regression models were fitted and odds ratios (ORs) with 95% CIs based on cluster-robust variances were calculated.

In a subgroup analysis of all post-SOP patients, we performed propensity score matching based on a logistic regression on SOP adherence with age, gender, BMI, diabetes, previous heart surgery, acute-on-chronic cardiomyopathy and AMI, using the nearest-neighbor algorithm without replacement and with a caliper width of 0.2. For outcome analysis, we used stratified Cox regression and logistic regression with cluster-robust variances.

R version 4.0.2 (R development Core team (2020). R: A Language and Environment for Statistical Computing) was used for all statistical analyses.

## 3. Standard Operating Procedure

**Figure 1:**
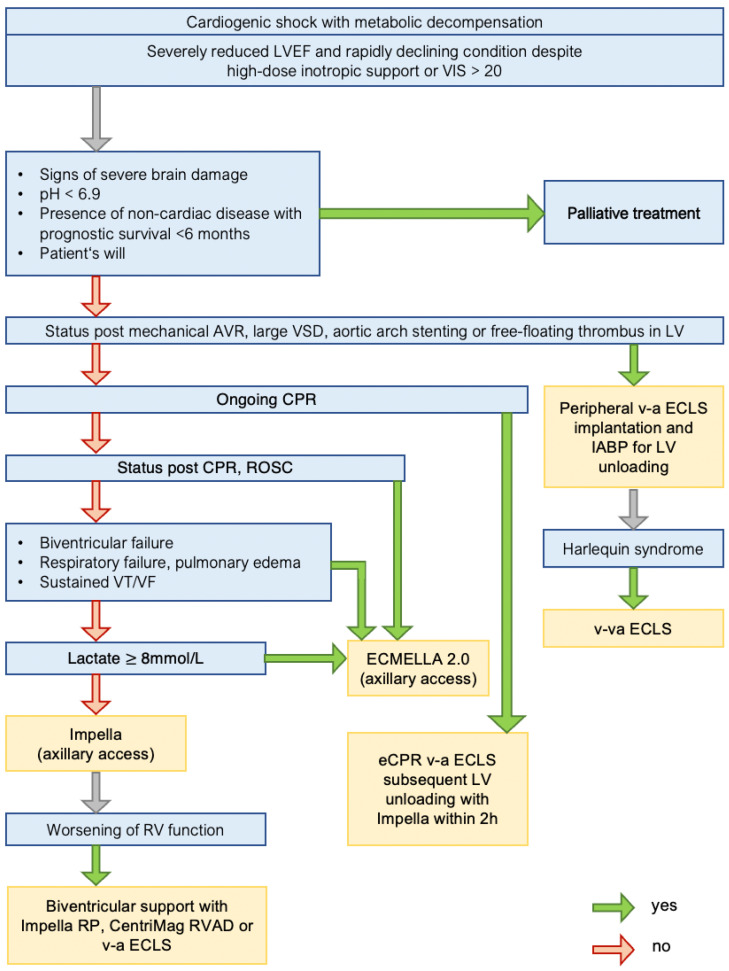
SOP for allocating patients in CS to a specific tMCS approach. AVR—aortic valve replacement. CPR—cardiopulmonary resuscitation. LVEF—left ventricular ejection fraction. ROSC—return of spontaneous circulation. RV—right ventricle. RVAD—right ventricular assist device. V-a ECLS—venoarterial extracorporeal life support. VF—ventricular fibrillation. VIS—vasoactive–inotropic score. VSD—ventricular septal defect. VT—ventricular tachycardia.

The SOP for allocating patients in CS to a specific tMCS approach is shown in Figure 1. According to the current guidelines [8], patients receiving medical treatment for CS are continuously monitored for indicators of hemodynamic deterioration. Particularly metabolic decompensation as evidence of an insufficient circulatory supply despite inotropic support and an increasing demand for vasopressor therapy are entry criteria for further escalation to tMCS in accordance with current guidelines.

First, conditions that would lead to a palliative care approach are excluded. Patients with ongoing cardiopulmonary resuscitation (CPR) are initially supported with a peripheral v-a ECLS to provide extracorporeal CPR (eCPR), followed by implantation of a percutaneous microaxial flow pump within two hours. Patients with ROSC after CPR; patients with biventricular failure, respiratory failure, ongoing ventricular tachycardia or fibrillation; and patients with a lactate level >8 mmol/L are supported with ECMELLA 2.0. This concept enables a combined MCS approach through a single arterial access, facilitating early mobilization and a bedside de-escalation strategy in these patients [16]. For the updated ECMELLA 2.1 concept, the ECLS venous drainage is inserted via the right internal jugular vein to facilitate unrestricted patient mobilization.

Patients in whom none of the above conditions apply are supported with a single-modality tMCS approach using a percutaneous microaxial flow pump (Impella 5.5 or previously 5.0 (Abiomed, Danvers, MA, USA)) inserted via an axillary artery.

Absolute contraindications for percutaneous microaxial flow pumps are limited to the presence of a mechanical aortic valve prosthesis and a free-floating left ventricular (LV) thrombus. Large ventricular septal defects are not a formal contraindication; however, the use of percutaneous microaxial flow pumps in such cases is complex and the benefit is questionable [17,18,19]. Therefore, large ventricular septal defects are listed as a contraindication in our SOP. A small marginal apical thrombus or smaller ventricular septal defects make percutaneous microaxial flow pump therapy challenging, but they do not constitute contraindications. Our first-line approach for percutaneous microaxial flow pump therapy is a graft-assisted access via the axillary artery to enable early mobilization in these patients. The SOP lists aortic arch stents as a contraindication for this approach. However, this scenario reflects a rare condition, and femoral access for an Impella CP is a viable alternative in these special cases.

Patients with absolute contraindications for these modified approaches are allocated to traditional peripheral v-a ECLS implantation.

## 4. Results

### 4.1. Study Cohort

Details of the study cohort are shown in Table 1. Between January 2018 and May 2021, 451 patients suffering from CS were scheduled for tMCS therapy and enrolled in the tMCS database. Prior to October 2019, 189 patients were treated with a tMCS approach (pre-SOP group); of these, 24 patients (11.4%) were allocated to a proactive palliative care approach. Following a revision of the SOP in October 2019, 232 patients received tMCS therapy (SOP group); of these, 6 patients (2.5%) were treated with a proactive palliative care approach (Figure S1). After propensity score matching, the cohort was reduced to 306 patients, yielding 153 pairs with 1:1 matching. 

The SOP group was further subdivided into a group where the SOP decision algorithm was followed (SOP adherent group, SOP-A, n = 120/230, 52.17%) and a second group with SOP derogations (SOP non-adherent group, SOP-NA, n = 110/230, 47.82%). Due to missing data, 2 of the 232 patients after the SOP implementation were excluded (Table 2). The subgroups were again propensity score-matched to ensure comparability, yielding 72 matched pairs in these subgroups. 

### 4.2. Outcome Analysis

#### 4.2.1. Unmatched Cohorts

In the unmatched cohort, the 30-day survival in the SOP group was 43.1% compared to 37.4% in the pre-SOP group (*p* = 0.282), and the 1-year survival was 28.2% vs. 26.1% (*p* = 0.726), respectively. Hospital discharge was 36.0% in the SOP group compared to 26.8% in the pre-SOP group (*p* = 0.198). The corresponding HR for the pre-SOP group was 1.13 (95% CI 0.90–1.42, *p* = 0.285).

#### 4.2.2. Matched Cohorts

**Figure 2:**
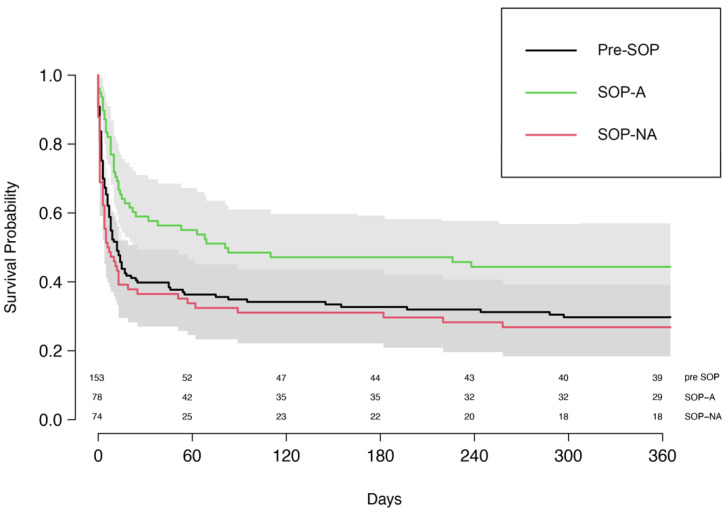
Kaplan–Meier curve of the matched study cohort, pre-SOP vs. SOP-A and SOP-NA.

After matching, the baseline values that were not part of the SOP decision algorithm were balanced between the groups (Supplementary Figure S2). In the matched groups, 30-day survival was 48.4% in the SOP group compared to 39.5% in the pre-SOP group, yielding an OR of 1.45 (95% CI [0.87, 2.40], *p* = 0.159). One-year survival was 33.1% vs. 27.6% (OR 1.33, 95% CI [0.75, 1.37], *p* = 0.334), respectively; see Figure 2. Hospital discharge was significantly higher in the SOP group compared to the pre-SOP group (41.7% vs. 29.7%). The corresponding OR for hospital discharge was 1.74 (95% CI 1.02–2.98, *p* = 0.043). The HR for the pre-SOP was 1.11 (95% CI [0.83, 1.47], *p* = 0.485).

#### 4.2.3. Matched Subgroups SOP-A and SOP-NA

**Figure 3:**
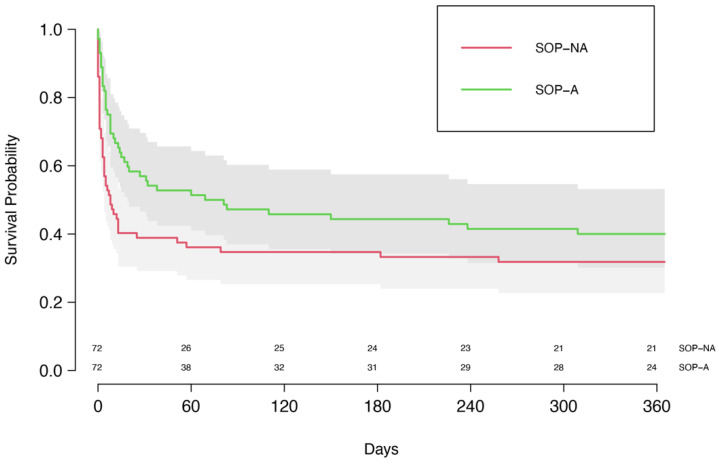
Kaplan–Meier curve of the matched study groups SOP-A and SOP-NA.

When comparing the matched SOP subgroups to further separate patients who were treated in accordance with the implemented SOP, 30-day survival was significantly higher in the SOP-A group when compared to the SOP-NA group (56.9% vs. 38.9%), with a corresponding OR of 2.21 (95% CI [1.01, 4.80], *p* = 0.044), Figure 3. One-year survival was 40.0% compared to 31.8% (OR 1.39, 95% CI [0.62, 3.13], *p* = 0.424), and hospital discharge was 47.1% in the SOP-A group compared to 32.5% in the SOP-NA group (OR 1.49, 95% CI [0.70, 3.19], *p* = 0.304). The corresponding HR for the SOP-A group was 0.69 (95% CI [0.46, 1.02], *p* = 0.063).

## 5. Discussion

Data from the retrospective propensity score-matched analysis presented here show that the implementation of and adherence to our SOP decision algorithm was associated with an increased 30-day survival. This improvement can be attributed to a structured and focused approach in patients with CS, a more appropriate patient selection for a specific tMCS approach, the combination therapies with more than one ECLS modality, and earlier and more effective LV unloading using microaxial flow pumps. 

### 5.1. Rationale of SOP Revision

CS causes a rapid deterioration of end-organ function leading to a life-threatening vicious circle [20]. Percutaneous microaxial flow pumps and v-a ECLS both provide circulatory support. While v-a ECLS allows for both circulatory and respiratory support, it increases LV afterload which reduces the chance of LV recovery [21,22]. Microaxial flow pumps provide circulatory support by active LV unloading, but no respiratory support is possible and sufficient right heart function is required. While percutaneous microaxial flow pumps improve ventricular recovery in patients suffering from cardiogenic shock [23], the maximum extent of support depends on the actual device (Impella 2.5, CP, 5.0 or 5.5) with the type-specific maximum flow limits inherent to the design. In the presence of respiratory and/or right ventricular failure, ECLS is necessary. V-a ECLS as a stand-alone tMCS strategy is limited by increasing adverse event rates with longer support times and the lack of LV unloading [24]. It was shown to be inferior to a combined approach using a percutaneous microaxial flow pump and v-a ECLS, the so-called ECMELLA concept [25]. Schrage et al. were able to demonstrate the superiority of a combined MCS approach with ECMELLA compared to isolated support with v-a ECLS in patients with acute myocardial infarction-associated cardiogenic shock, despite the increased complication rate with increased bleeding events in the group treated with microaxial flow pumps. Several studies and meta-analyses support this approach [26,27,28]. Possible complications of percutaneous microaxial flow pump devices have to be taken into account; however, the survival benefit offered by the combined ECMELLA approach puts this into perspective. Lorusso et al. recently summarized the current evidence for LV unloading and recommended LV unloading as part of an effective tMCS therapy whenever possible [29].

In this sense, Impella and v-a ECLS are not competing but complementing strategies. The key question is when to escalate from a single-modality approach to a complementary tMCS strategy—such as the ECMELLA concept. In a previous study, we were able to show that the level of support required should be driven by the stage of shock [30]. Patients with a lactate level above 8 mmol/L and/or post-resuscitation prior to implantation of Impella 5.0 or 5.5 had a decreased 30-day survival rate when treated with Impella alone. In these cases, the required flow rates necessary for a reversal of end-organ damage appear beyond the limits of an isolated Impella approach, and consequently, our SOP was adapted to primarily allocate such patients to ECMELLA. 

### 5.2. Outcome

A crude comparison of patients treated before and after implementation of the new SOP yielded no significant change in hospital discharge, 30-day survival or 1-year survival. After propensity score matching, the likelihood of hospital discharge was 74% higher for patients treated after the implementation of our SOP. Furthermore, patients treated according to the SOP were less likely to die within the first year after the event, even though the HR of 0.69 slightly failed to reach statistical significance.

Introducing improvements in technology and learning from clinical research for the treatment of CS in daily practice is challenging. A delay of up to 17 years is assumed for the transfer of knowledge from scientific evidence into clinical practice [31,32,33]. A mere 50% adherence to the revised SOP within less than two years leaves ample room for further improvement but is not unexpected. This can hopefully be improved upon further by highlighting the positive effects on outcomes. To evaluate the impact of the updated support algorithms stipulated by the SOP, rather than just the impact of the SOP implementation per se, we differentiated between patients who were actually treated according to the SOP (SOP-A) and those who were not (SOP-NA). Patients who were treated according to the SOP had a more than 2-fold higher likelihood of 30-day survival when compared to those not treated according to the SOP.

Concerning the other studied endpoints, hospital discharge and 1-year survival tended to be improved in the SOP-A group; however, due to the low number of cases subsequent to propensity score matching, they failed to achieve statistical significance. 

In summary, treating patients according to our SOP significantly increases the 30-day survival. We postulate that this improvement in patient outcomes was achieved mainly through enhanced patient selection and inclusion into standardized treatment algorithms. A lower VIS and a lower lactate level in the SOP group likely indicate an earlier treatment with a tMCS approach. We consider this finding one of the key results of the implementation of the revised SOP, which is also reflected in current guidelines [8].

### 5.3. Observed Cohort and Comparative Evaluation

As a tertiary specialized cardiovascular center, we provide care to patients referred by numerous surrounding healthcare providers. Due to this highly specialized status, our patient portfolio differs from that of most other cardiological or cardiosurgical departments, and we have a disproportionately high number of patients in advanced stages of shock, but relatively few patients with new-onset cardiovascular events and early stages of CS. Furthermore, in most of these patients, a first-line standard approach—whether primary percutaneous coronary intervention or tMCS— failed, which is why a large proportion of them was scheduled to undergo urgent or emergent cardiac surgery.

Patients who had to be allocated to tMCS within seven days of cardiac surgery were classified as PCCS. In this group with a well-documented high level of mortality, it is nearly impossible to discriminate whether the need for tMCS is due to their underlying disease, the postcardiotomy syndrome or both [4]. Because of the heterogeneous nature of this patient population, such cohorts are frequently not included in outcome analyses, unlike in the study presented here. We explicitly decided to keep PCCS patients in the analysis to reflect the impact of the SOP on all patients requiring tMCS. Bearing in mind the tremendously high mortality rates of PCCS patients, an overall 30-day survival of 41% is an encouraging result.

### 5.4. Limitations

Our study has several limitations. This is a retrospective analysis conducted in a single, high-volume center, and the aspect of predominantly treating patients who failed to respond to initial treatment elsewhere must be considered. Consequently, the heterogeneity of the patient cohort is not negligible, and therefore, differentiated conclusions are hard to draw. Furthermore, since an overwhelming majority of the patients were transferred to our hospital by another healthcare provider, time-to-treat analyses are hard to perform. The retrospective design of the study entails numerous known limitations; however, propensity score matching of the patient groups ensured comparability. Some of our findings failed to reach statistical significance, which is most likely due to the limited number of patients. The presented SOP is based on published studies, an internally developed best clinical practice and expert opinions. Furthermore, this study presents an SOP as an encompassing treatment algorithm, and thereby no conclusion on the significance of single aspects or MCS devices can be drawn.

## 6. Conclusions

The SOP presented here offers a simple and versatile approach for patients in CS secondary to various etiologies. In this propensity score-matched analysis, treating patients according to the SOP decision algorithm was associated with an increased 30-day survival and a trend towards a decreased likelihood to die within the first year after the event. The results support undertaking larger, prospective trials to confirm the outcome improvements associated with the implementation of this SOP.

## Figures and Tables

**Table 1:** Pre-SOP vs. SOP.

Variable	Unmatched Cohort (n = 421)			Matched Groups (n = 306)		
	Pre-SOP (n = 189)	SOP (n = 232)	*p*	Pre-SOP (n = 153)	SOP (n = 153)	*p*
Age (years)	60.12 {13.99}	61.94 {13.01}	0.165	60.50 {14.45}	60.14 {13.79}	0.842
Sex (male)	146 (77.2)	173 (74.6)	0.600	121 (79.1)	113 (73.9)	0.345
BMI	27.59 {5.72}	28.37{6.64}	0.221	27.99 {5.83}	27.64 {5.37}	0.593
Diabetes	61 (32.3)	70 (31.0)	0.859	50 (32.7)	44 (28.8)	0.536
CKD	45 (24.3)	74 (32.9)	0.073	44 (28.8)	43 (28.1)	1.000
MAP (mmHg)	66.00 [57.50, 75.00]	69.00 [62.00, 78.00]	0.017	66.00 [58.00, 76.00]	68.00 [61.00, 77.00]	0.12
Heart rate (bpm)	96.00 [82.00, 112.50]	93.00 [78.00,105.00]	0.136	97.00 [82.00, 111.00]	93.00 [77.00, 104.00]	0.178
Lactate (mmol/L)	6.11 [2.78,10.99]	4.78 [1.72,10.1]	0.014	6.05 [2.77,10.57]	4.22 [1.44,10.19]	0.258
pH	7.32 [7.25, 7.39]	7.31 [7.23, 7.39]	0.352	7.32 [7.25, 7.39]	7.31 [7.22, 7.40]	0.142
VI score	32.00 [13.00, 48.90]	18.88 [8.00, 41.35]	0.001	30.70 [13.00, 51.98]	18.00 [7.61, 40.75]	0.01
RRT prior to tMCS	21 (11.2)	27 (11.7)	1.000	20 (13.2)	19 (12.5)	0.982
Cause of CS						
AMICS	34 (18.0)	24 (10.3)	0.034	21 (13.7)	22 (14.4)	1.000
Acute-on-chronic CMP	56 (29.6)	90 (38.8)	0.063	49 (32.0)	51 (33.3)	0.903
PCCS	80 (42.3)	100 (43.1)	0.951	69 (45.1)	67 (43.8)	0.912
Other	18 (10.1)	18 (7.8)	0.502	14 (9.2)	13 (8.5)	0.840
Previous cardiac arrest	71 (37.6)	58 (25.0)	0.007	48 (31.4)	45 (29.4)	0.731
Duration of CPR (min)	20.00 [10.00, 58.75]	20.00 [10.00, 50.75]	0.459	17.50 [10.00, 40.00]	19.50 [10.50, 53.75]	0.401
eCPR	30 (18.5)	36 (20.9)	0.667	21 (16.3)	26 (23.4)	0.22
Mechanical aortic valve	2 (1.2)	0 (0)	0.465	2 (1.5)	0 (0.0)	0.559
Aortic arch stent	1 (0.6)	4 (2.3)	0.385	1 (0.8)	1 (0.9)	1.000
Free-floating LV thrombus	0 (0)	1 (0.6)	1.000	0 (0.0)	1 (0.9)	0.928
MCS type						
v-a ECLS	139 (73.5)	109 (47.0)	<0.001	109 (71.2)	70 (45.8)	<0.001
Impella	26 (13.8)	61 (26.3)	0.002	20 (13.1)	41 (26.8)	0.004
ECMELLA	24 (12.7)	62 (26.7)	0.001	23 (15.0)	42 (27.5)	0.012

AMICS—acute myocardial infarction-associated cardiogenic shock; BMI—body mass index; CKD—chronic kidney disease; CMP—cardiomyopathy; CS—cardiogenic shock; eCPR—extracorporeal cardiopulmonary resuscitation; MAP—mean arterial pressure; PCCS—postcardiotomy syndrome cardiogenic shock; RRT—renal replacement therapy; tMCS—temporary mechanical circulatory support; v-a ECLS—venoarterial extracorporeal life support; VI score—vasoactive–inotropic score. Data are presented as number (%) or mean {SD}. Data followed by square brackets show the median with interquartile range. Grey highlighted fields indicate significantly differing results, assumed by a *p*-value < 0.05.

**Table 2:** SOP-A vs. SOP-NA.

Variable	Unmatched Cohort (n = 230)			Matched Groups (n = 144)		
	SOP-A (n = 120)	SOP-NA (n = 110)	*p*	SOP-A (n = 72)	SOP-NA (n = 72)	*p*
Age (years)	59.63 {13.06}	64.44 {12.53}	0.005	62.07 {12.17}	62.58 {12.38}	0.802
Sex (male)	97 (80.8)	74 (67.3)	0.028	56 (77.8)	57 (79.2)	1.000
BMI	28.11 {7.24}	28.71 {5.95}	0.506	28.72 {7.67}	28.88 {5.64}	0.888
Diabetes	37 (31.6)	32 (29.9)	0.894	24 (33.3)	23 (31.9)	1.000
CKD	39 (33.6)	35 (32.7)	0.998	23 (31.9)	25 (34.7)	0.860
MAP (mmHg)	71.00 [63.00, 77.00]	68.00 [60.50, 77.50]	0.571	69.00 [63.00, 76.00]	67.00 [59.25, 81.00]	0.661
Heart rate (bpm)	93.00 [79.00, 105.00]	94.00 [78.00, 105.50]	0.698	91.00 [77.00, 105.00]	98.50 [87.00, 108.00]	0.335
Lactate (mmol/L)	2.99 [1.44, 6.13]	7.55 [3.77, 10.93]	<0.001	3.05 [1.55, 6.05]	8.55 [4.17, 10.88]	<0.001
pH	7.34 [7.26, 7.40]	7.29 [7.20, 7.37]	0.018	7.33 [7.24, 7.40]	7.29 [7.20, 7.37]	0.087
VI score	18.00 [8.89, 37.75]	21.60 [8.00, 42.25]	0.504	19.00 [9.79, 40.00]	26.45 [10.50, 51.30]	0.331
RRT prior to tMCS	10 (8.3)	16 (14.7)	0.193	8 (11.1)	14 (19.7)	0.232
Cause of CS						
AMICS	17 (14.2)	7 (6.4)	0.086	7 (9.7)	5 (6.9)	0.763
Acute-on-chronic CMP	50 (41.7)	38 (34.5)	0.330	31 (43.1)	27 (37.5)	0.610
PCCS	48 (40.0)	52 (47.3)	0.328	29 (40.0)	32 (44.4)	0.742
Other	5 (4.1)	13 (11.8)		5 (7.2)	8 (11.2)	0.356
Previous cardiac arrest	31 (25.8)	27 (24.5)	0.942	17 (23.6)	16 (22.2)	1.000
Duration of CPR (min)	20.00 [10.00, 51.25]	21.50 [10.50, 60.00]	0.814	35.00 [10.00, 57.75]	14.00 [6.00, 51.25]	0.279
eCPR	11 (15.5)	25 (24.8)	0.176	6 (14.6)	19 (27.9)	0.133
Mechanical aortic valve	0 (0.0)	0 (0.0)	NaN	0 (0.0)	0 (0.0)	NaN
Aortic arch stent	3 (4.2)	1 (1.0)	0.383	2 (4.9)	1 (1.5)	0.653
Free-floating LV thrombus	1 (1.4)	0 (0.0)	0.859	1 (2.4)	0 (0.0)	0.797
MCS type				MCS type		
v-a ECLS	8 (6.7)	101 (91.8)	<0.001	6 (8.3)	68 (94.4)	<0.001
Impella	50 (41.7)	9 (8.2)	<0.001	32 (44.4)	4 (5.6)	<0.001
ECMELLA	62 (51.7)	0 (0)	<0.001	34 (47.2)	0 (0.0)	<0.001

AMICS—acute myocardial infarction-associated cardiogenic shock; BMI—body mass index; CKD—chronic kidney disease; CMP—cardiomyopathy; CS—cardiogenic shock; eCPR—extracorporeal cardiopulmonary resuscitation; MAP—mean arterial pressure; PCCS—postcardiotomy syndrome cardiogenic shock; RRT—renal replacement therapy; stMCS—short-term mechanical circulatory support; v-a ECLS—venoarterial extracorporeal life support; VI score—vasoactive–inotropic score. Data are presented as number (%) or mean {SD}. Data followed by square brackets show the median with interquartile range. Grey highlighted fields indicate significantly differing results, assumed by a *p*-value < 0.05.

## Data Availability

The raw data supporting the conclusions of this article will be made available by the authors, without undue reservation.

## Supplementary Materials

The following supporting information can be downloaded at: https://www.mdpi.com/article/10.3390/life12111931/s1, Figure S1: Patient selection; Figure S2: Covariate balance plot.
