# Supplementary figures and images for: Improving Survival in Cardiogenic Shock—A Propensity Score-Matched Analysis of the Impact of an Institutional Allocation Protocol to Short-Term Mechanical Circulatory Support
Source: Life (Basel). 2022 Nov 19;12(11):1931. doi: 10.3390/life12111931 (PMC9692664; doi:10.3390/life12111931)

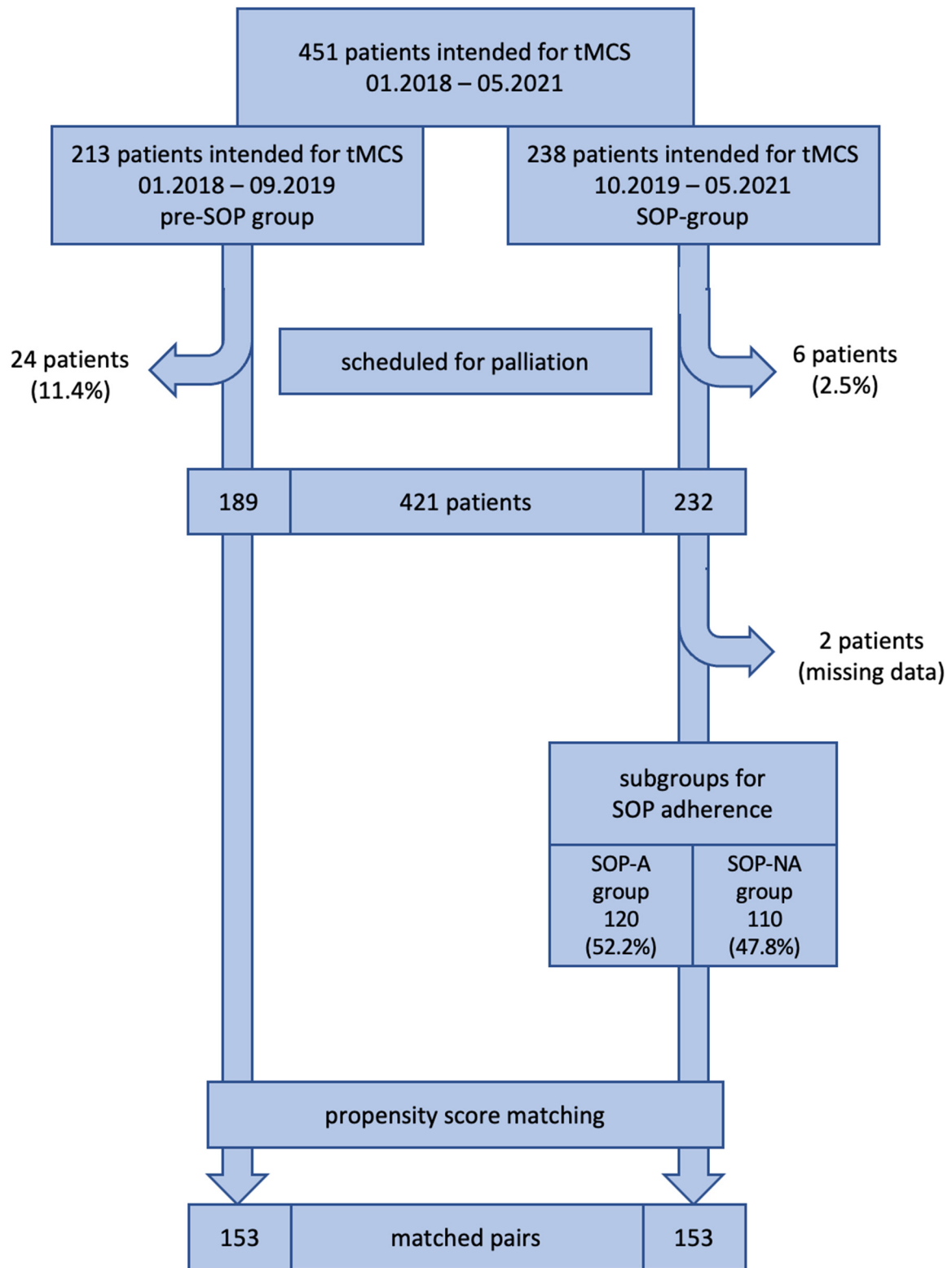

**Figure S1.** Patient selection.

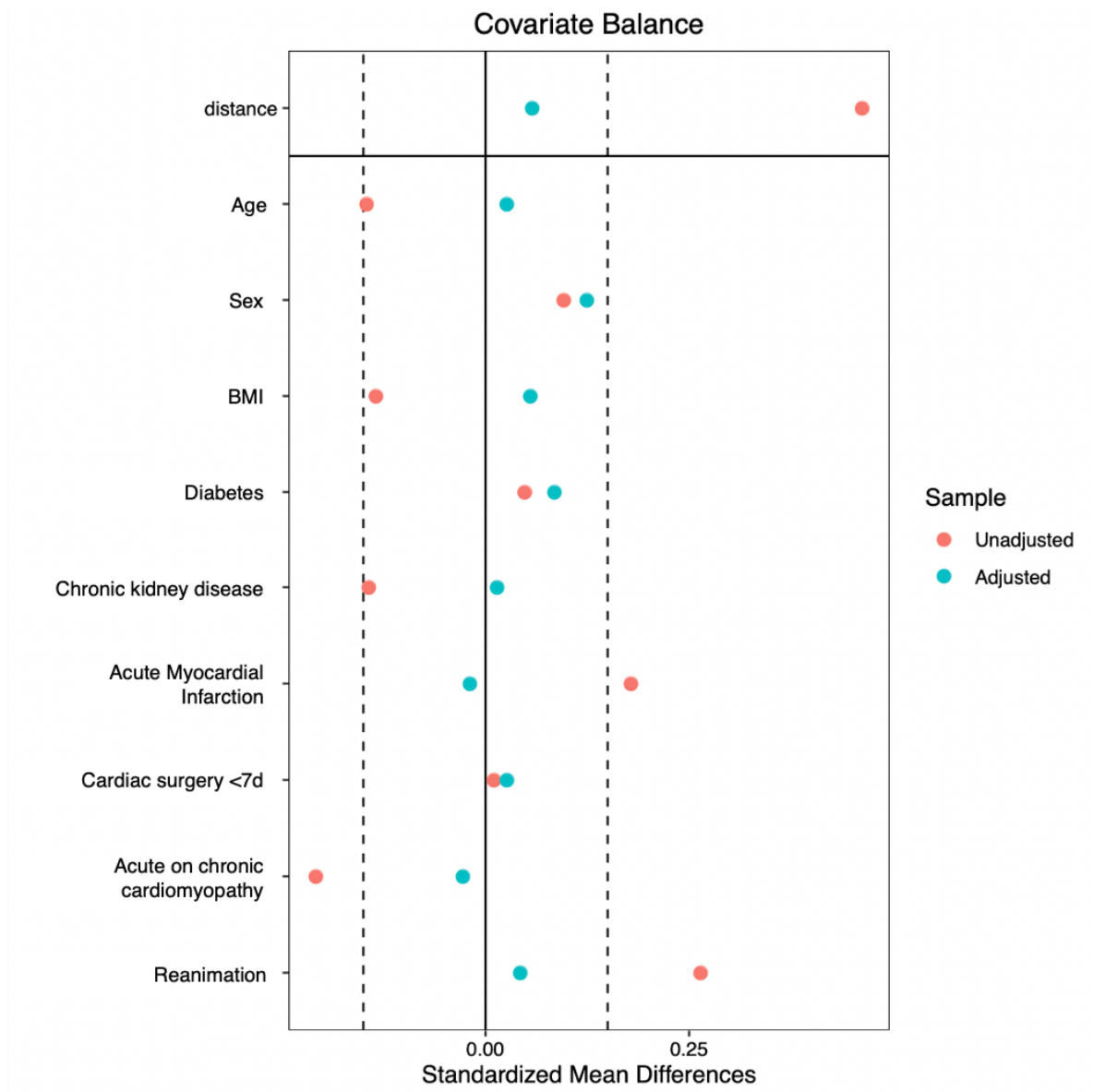

**Figure S2.** Covariate balance plot.

Supplement: Supplementary file 1 [file life-12-01931-s001.zip › life-1990335-supplementary.pdf]
